# Reduction in Renal Relapse and Preservation of Long‐Term Kidney Function After Lupus Low Disease Activity in Patients With Lupus Nephritis

**DOI:** 10.1002/acr.25611

**Published:** 2025-11-21

**Authors:** Chak Kwan Cheung, Desmond Y. H. Yap, Ka Lai Lee, Philip H. Li, Iris Y. K. Tang, Chak Sing Lau, Shirley C. W. Chan

**Affiliations:** ^1^ Queen Mary Hospital The University of Hong Kong Pokfulam Hong Kong; ^2^ Pamela Youde Nethersole Eastern Hospital Chai Wan Hong Kong

## Abstract

**Objective:**

Lupus low disease activity state (LLDAS) is a validated treatment target in systemic lupus erythematosus (SLE), but limited studies have explored the role of LLDAS in lupus nephritis (LN). This study aims to investigate the frequency and predictors of LLDAS attainment and its benefit on LN relapse and renal function preservation in patients with LN.

**Methods:**

Patients with LN during 2010 to 2020 in Queen Mary Hospital and Pamela Youde Nethersole Eastern Hospital were included in the discovery cohort and validation cohort, respectively. Complete renal response (CRR), partial renal response (PRR), LLDAS, and Definition Of Remission In SLE (DORIS) remission were assessed at 12 months. Regression analysis was performed to identify risk factors of LN relapse. Receiver operating characteristic (ROC) curves were used to evaluate target attainment and long‐term kidney function.

**Results:**

A total of 245 patients with LN (discovery cohort n = 143 and validation cohort n = 102) were included. At 12 months, 57 of 143 (40%), 14 of 143 (10%), 70 of 143 (49%), and 15 of 143 (10%) patients achieved CRR, PRR, LLDAS, and DORIS remission, respectively. Attainment of both CRR/PRR and LLDAS at 12 months was associated with best relapse‐free survival (*P* < 0.001). Multivariate analysis showed independent association of CRR/PRR and LLDAS with LN relapse risk reduction (CRR/PRR: hazard ratio [HR] 0.31, *P* = 0.007; LLDAS: HR 0.38, *P* = 0.029). LLDAS attainment predicts renal function preservation with satisfactory performance in both the discovery and validation cohorts (area under the curve of the ROC 0.71).

**Conclusion:**

LLDAS is an attainable target in LN comparable to CRR/PRR. Attainment of both targets is associated with additional benefits on relapse risk reduction. Early LLDAS attainment is associated with renal function preservation.

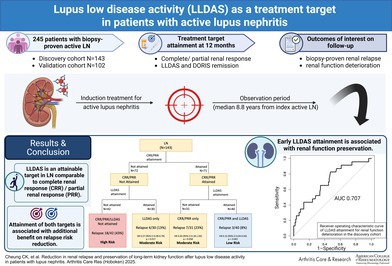

## Graphical abstract

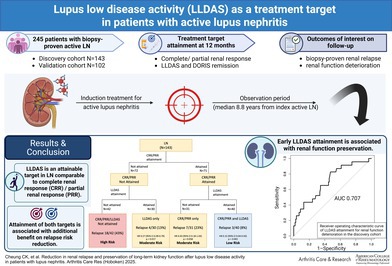

## INTRODUCTION

Lupus nephritis (LN) is an important manifestation affecting 50% to 60% of patients with systemic lupus erythematosus (SLE).^1^, ^2^ Patients with LN are at risk of disease relapse, irreversible renal damage, and subsequent progression to end‐stage renal disease (ESRD).^3^ Given the association between proteinuria and long‐term renal outcomes, current treatment targets are primarily based on improvement in proteinuria and preservation of renal function as measured by serum creatinine or estimated glomerular filtration rate (eGFR).^4^, ^5^ Despite the usefulness of current treatment targets, a considerable number of patients experience LN relapse.^3^ Other factors are believed to influence overall outcomes in LN, including serological activities and nonrenal disease activities.^6^, ^7^

**SIGNIFICANCE & INNOVATIONS**

- Lupus low disease activity state (LLDAS) is an attainable target associated with reduced renal relapse in lupus nephritis (LN).
- Attainment of LLDAS in addition to complete and partial renal response confers extra benefit on LN relapse reduction.
- LLDAS attainment predicts long‐term renal protection.

Lupus low disease activity state (LLDAS) is a validated treatment target associated with improved patient outcomes, including reduced risks of flare and prevention of organ damage in SLE.^8^ LLDAS encompasses five important domains related to disease and immunosuppressive treatment. Prior studies have shown a lower LLDAS attainment among patients with SLE and renal involvement, but studies to investigate the role of LLDAS in patients with LN remain limited.^9^, ^10^, ^11^ A more recent study on pediatric patients with LN demonstrated a beneficial role of LLDAS in the reduction of risk of LN relapse and damage accruals.^12^ Dedicated studies are needed to ascertain the benefit of LLDAS attainment and to compare its performance with current LN treatment targets.

Using longitudinal clinical data from an inception cohort of Chinese patients with LN from two tertiary hospitals in Hong Kong, this study aimed to investigate the frequency of LLDAS attainment and its potential benefit on LN relapse risk reduction and kidney function preservation in comparison with conventional renal response criteria. The study also evaluated clinical predictors of LLDAS attainment and LN relapse.

## PATIENTS AND METHODS

### Study population and data collection

#### Discovery cohort

The discovery cohort comprised 143 patients with biopsy‐proven active LN (incident or prevalent cases) during the period of 2010 to 2020 in Queen Mary Hospital. Data from the discovery cohort were used to evaluate the frequency and predictors of LLDAS attainment as well as the effect of LLDAS attainment on the risk of relapse and kidney function preservation. Baseline demographics including sex and age of disease onset were collected. The baseline SLE Disease Activity Index 2000 (SLEDAI‐2K) of all patients was documented.^13^ All patients were observed regularly at intervals no longer than every 4 months for clinical and laboratory test monitoring, with additional visits decided by treating physicians depending on clinical needs. Renal biopsies were evaluated by pathologists according to the International Society of Nephrology/Renal Pathology Society 2003 or 2018 classifications.^14^ Blood parameters, including serum albumin, creatinine, eGFR, serum complements, and anti–double‐stranded DNA (dsDNA) titer, were documented at every visit. eGFR was calculated using the four‐variable modification of diet in renal disease formula.^15^ The urine profile included 24‐hour urine protein or the urine protein‐to‐creatinine ratio (UPCR). Deidentified data from the current study will be made available upon reasonable request to the corresponding author.

The treatment regimen was decided by treating physicians (rheumatologists or nephrologists specialized in SLE) based on histologic subtypes, renal function deterioration, extrarenal activities, other comorbidities, and patient preferences. Treatment was made up of an induction phase, which included high‐dose glucocorticoids and immunosuppressants, followed by maintenance therapy. First‐line induction treatment for proliferative LN included mycophenolate mofetil (MMF) or cyclophosphamide, whereas calcineurin inhibitors or azathioprine were used in certain patients based on other considerations (eg, pregnancy wish) or in patients with nonproliferative subtypes.

#### Validation cohort

An independent validation cohort was made up of 102 patients with biopsy‐proven active LN between 2010 and 2020 from Pamela Youde Nethersole Eastern Hospital. All patients in the validation cohort had regular follow‐up visits to monitor treatment response and LN relapse.

### Definitions of criteria

Renal response and LLDAS were assessed 12 months after the biopsy date. Renal response criteria included complete renal response (CRR), partial renal response (PRR), and no response (NR). CRR was defined as proteinuria ≤0.5 g/day with normal or near‐normal eGFR (defined as within 10% of 90 mL/min/1.73m^2^), PRR was defined as a reduction in proteinuria by ≥50% but >0.5 g/day with normal or near‐normal eGFR, and NR was defined as not meeting either CRR or PRR. Reduced eGFR beyond near‐normal range will be classified as NR regardless of the proteinuric response.^16^

LLDAS was defined as meeting all of the following criteria: (1) SLEDAI‐2K score ≤4 with no activity in major organ systems and no hemolytic anemia or gastrointestinal activity, (2) no new lupus disease activity compared with the previous assessment, (3) a Safety of Estrogens in Lupus Erythematosus National Assessment–SLEDAI Physician Global Assessment (PGA) (scale 0–3) score ≤1, (4) a current prednisolone dose ≤7.5 mg/day, and (5) standard maintenance doses of immunosuppressants and approved biologic agents.^8^ Definition Of Remission In SLE (DORIS) remission was defined as meeting all of the following criteria: (1) clinical SLEDAI‐2k score of 0, (2) PGA < 0.5, (3) a current prednisolone dose ≤5 mg daily, and (4) stable doses of immunosuppressants and biologics.^17^

### Study endpoints

The definition of LN relapse in the current study was adapted from similar or landmark trials.^18^ LN relapse was suspected when there was worsening of proteinuria and/or urinary sediments and/or serum creatinine, combined with clinical judgment by the attending rheumatologist/nephrologist, and confirmed with renal biopsy. Worsening of proteinuria was defined as an increase of the UPCR or 24 hour urine protein to ≥1 mg/mg or 1 g/day in patients with ≤0.5 g at end of induction; or ≥2 mg/mg or 2 g/day in patients with >0.5 g at the end of induction. Urinary sediments included hematuria, pyuria or presence of hyaline, and granular or cellular casts. Worsening of serum creatinine was defined as increase of >25% from the lowest value after the end of induction therapy. All patients had attained clinically meaningful treatment response (defined as a reduction of 24‐hour urinary protein or the equivalent of UPCR by 50% if the baseline 24‐hour urinary protein was <3 g or to <3 g if baseline proteinuria was >3 g) before renal relapse. All LN relapses were assessed after the initial 12 months.

Deterioration of renal function was defined as sustained impairment with doubling of baseline serum creatinine. Chronic kidney disease (CKD) stages were defined according to the Kidney Disease: Improving Global Outcomes definition based on eGFR.^18^ CKD stage one was defined as eGFR ≥90 mL/min/1.73m^2^, stage two was 60 to 89 mL/min/1.73m^2^, stage three was 30 to 59 mL/min/1.73m^2^, stage four was 15 to 29 mL/min/1.73m^2^, and stage five was <15 mL/min/1.73m^2^. Baseline demographics, laboratory parameters, and serologic markers along with the choice of induction and maintenance therapy were included in the association analyses of LLDAS attainment at 12 months and LN relapse.

### Statistical analysis

The sample size required to achieve 85% power with a 5% margin of error was calculated to be 133 based on the assumption of a 20% LN relapse rate in previous publications. Continuous variables were expressed as median and interquartile range (IQR). Categorical variables were expressed as percentages. Logistic regression analysis was performed to identify predictors associated with LLDAS, and the results were reported as odds ratios (ORs) with 95% confidence intervals (CIs). Time to LN relapse between LLDAS attainment and renal response criteria was compared using the Kaplan‐Meier curve and log‐rank test. Cox regression analysis was performed to identify factors associated with LN relapse. Variables with a *P* value <0.10 in the univariable analysis were included in the multivariable analysis, and variables with a *P* value <0.05 in the multivariable regressions were considered statistically significant. Receiver operating characteristic (ROC) curves with area under the curve (AUC) were used to evaluate the performance of LLDAS at predicting renal function deterioration. Statistical analysis was performed on the statistical software IBM SPSS Statistics (version 28.0.1; IBM Co). A two‐sided *P* value <0.05 was considered statistically significant. Survival curves were plotted with R software (version 4.2.2). The study was approved by the Institutional Review Board of the University of Hong Kong/Hospital Authority of Hong Kong West Cluster.

## RESULTS

The discovery cohort was made up of 143 patients with LN with a median disease duration of 14 years (IQR 7.0–20.0 years). Most patients were female (131 of 143; 92%) and had either class IV ± V (68 of 143; 48%) or class III ± V (38 of 143; 27%) LN (Table 1). Active serological activity was found in most patients, including 124 of 143 (87%) and 116 of 143 (81%) patients with hypocomplementemia and elevated anti‐dsDNA titer, respectively. A small number of patients (8 of 143; 6%) had inactive serology, and renal biopsy was performed because of proteinuria and/or urinary sediments and/or impaired renal function. Most patients had preserved renal function (83 of 143; 58%) and subnephrotic‐range proteinuria (116 of 143; 81%). Extrarenal disease (as measured by SLEDAI) occurred in 68 of 143 (48%) patients. Mucocutaneous (41 of 143, 29%), hematologic (25 of 143, 17%), and musculoskeletal systems (6 of 143, 4%) were the most common extrarenal domain with clinical disease activity at baseline. The median follow‐up duration was 8.8 years (IQR 6.0–10.5 years), and 32 patients developed LN relapse.

**Table 1:** Comparison of clinical characteristics of the discovery and validation cohorts*

Baseline characteristics	Discovery cohort (n = 143)	Validation cohort (n = 102)	*P* value
Sex (female), n/N (%)	131/143 (92)	89/102 (87)	0.267
Age at SLE onset, median (IQR), years	28 (20–35)	30 (18–39)	0.495
Disease duration, median (IQR), years	14 (7–20)	6 (1–12)	<0.001
Follow‐up duration, median (IQR), years	8.8 (6.0–10.5)	9.5 (6.4–10.0)	0.013
ISN/RPS LN classification, n/N (%)			
Class I/II	12/143 (8)	16/102 (16)	0.077
Class III (± V)	38/143 (27)	33/102 (32)	0.326
Class IV (± V)	68/143 (48)	40/102 (40)	0.195
Class V	25/143 (18)	13/102 (13)	0.313
24hUP (g) or UPCR (mg/mg), median (IQR)	1.6 (1.2–2.3)	2.1 (1.2–3.8)	0.006
>3 g/day, n/N (%)	27/143 (19)	34/102 (33)	0.010
Serum albumin, median (IQR), g/L	32 (28–35)	31 (26–35)	0.032
Serum creatinine, median (IQR), μmol/L	64 (52–87)	70 (60–93)	0.041
eGFR, median (IQR), mL/min/1.73m^2^	98 (67–123)	92 (67–110)	0.295
CKD, n/N (%)			
CKD1	83/143 (58)	52/102 (51)	0.273
CKD2	35/143 (25)	28/102 (28)	0.599
CKD3	19/143 (13)	20/102 (20)	0.182
CKD4	3/143 (2)	1/102 (1)	0.321
CKD5	3/143 (2)	1/102 (1)	0.769
Disease factors, median (IQR)			
SLEDAI score	8 (8–11)	8.5 (8–11)	0.237
PGA score	3 (2–3)	3 (2–3)	0.246
Immunologic factors, n/N (%)			
Low C3/C4	124/143 (87)	88/102 (88)	0.724
Anti‐dsDNA	116/ 143 (81)	93/102 (91)	0.028
Anti‐Sm	25/136 (18)	12/102 (12)	0.163
Anti‐Ro	67 (49)	46/102 (45)	0.524
Anti‐La	15/136 (11)	8/102 (8)	0.410
Anti‐RNP	45/136 (33)	23/102 (23)	0.075
Induction agent			
GC, n/N (%)	142/143 (99)	100/102 (98)	0.572
GC dose, median (IQR), mg	40 (30–50)	30 (25–45)	0.040
MMF, n/N (%)	111/143 (78)	66/102 (65)	0.026
CTX, n/N (%)	4/143 (3)	5/102 (5)	0.388
AZA, n/N (%)	14/143 (10)	17/102 (17)	0.110
CNI, n/N (%)	7/143 (5)	13/102 (13)	0.027
HCQ, n/N (%)	78/143 (55)	71/102 (70)	0.017
Biologics, n/N (%)^a^	5/143 (4)	0/102 (0)	0.078
Maintenance agent, n/N (%)			
MMF	107/143 (75)	49/102 (48)	<0.001
AZA	19/143 (13)	31/102 (30)	0.001
CNI	11/143 (8)	20/102 (20)	0.006
HCQ	85/143 (59)	70/102 (69)	0.115

* 24hUP, 24‐hour urine protein; AZA, azathioprine; C3, complement 3; C4, complement 4; CKD, chronic kidney disease; CNI, calcineurin inhibitor; CTX, cyclophosphamide; dsDNA, double‐stranded DNA; eGFR, estimated glomerular filtration rate; GC, glucocorticoid; HCQ, hydroxychloroquine; IQR, interquartile range; ISN/RPS, International Society of Nephrology/Renal Pathology Society; LN, lupus nephritis; MMF, mycophenolate mofetil; PGA, Physician Global Assessment; SLE, systemic lupus erythematosus; SLEDAI, SLE Disease Activity Index; Sm, Smith; UPCR, urine protein‐to‐creatinine ratio.

^a^ Biologics included rituximab and belimumab; all biologics were given with background MMF.

### LLDAS is an attainable treatment target comparable to CRR/PRR in patients with LN

The frequency and predictors of LLDAS attainment were analyzed in the discovery cohort. At 12 months, 57 of 143 (40%) and 14 of 143 (10%) patients in the discovery cohort achieved CRR and PRR, respectively. LLDAS and DORIS remission attainment was observed in 70 of 143 (49%) and 15 of 143 (9.1%) patients, respectively. Among 71 patients who attained CRR/PRR at 12 months, 40 of 71 (56%) also attained LLDAS. The remaining 42 of 143 (29%) patients in the discovery cohort failed to attain CRR/PRR or LLDAS at 12 months.

The fulfillment of each of the five LLDAS criteria was further assessed. At 12 months, the most frequently fulfilled criteria included tolerated standard therapy (140 of 143, 98%), no new disease activity (132 of 143, 92%), and PGA ≤1 (132 of 143, 92%). Only 106 of 143 (74%) and 88 of 143 (62%) of patients attained a prednisolone dose of ≤7.5 mg/day and SELDAI ≤ 4 without major organ involvement, respectively.

Baseline predictors for LLDAS attainment at 12 months in patients with active LN were evaluated in association analysis (Table 2; Supplementary Table S1). In the multivariable analysis, anti‐Sm positivity was a significant negative predictor of LLDAS attainment (adjusted OR 0.33; 95% CI 0.13–0.86, *P* = 0.024). Other variables showed no association with LLDAS attainment, including age, sex, histologic class, proteinuria, serum creatinine, and choice of induction agents.

**Table 2:** Predictors of lupus low disease activity state attainment at 12 months in the discovery cohort*

Baseline characteristics	Univariable analysis		Multivariable analysis	
	OR (95% CI)	*P* value	OR (95% CI)	*P* value
Female sex	1.38 (0.42–4.57)	0.599	–	–
Age at SLE onset, years	1.00 (0.97–1.03)	0.919	–	–
Prior history of LN	0.70 (0.36–1.35)	0.281	–	–
ISN/RPS LN classes				
Class III (± V)	Ref	Ref	–	–
Class IV (± V)	0.77 (0.40–1.97)	0.771	–	–
Class V	0.92 (0.34–2.53)	0.877	–	–
24hUP (g) or UPCR (mg/mg)	0.95 (0.78–1.17)	0.650	–	–
≥3 g	0.66 (0.28–1.55)	0.345	–	–
Serum albumin, g/L	1.05 (0.99–1.12)	0.136	–	–
Serum creatinine, μmol/L	1.00 (0.99–1.00)	0.221	–	–
eGFR, mL/min/1.73m^2^	1.00 (0.99–1.01)	0.795	–	–
Immunologic factors				
Low C3	0.42 (0.16–1.12)	0.085	0.47 (0.17–1.31)	0.147
Low C4	0.88 (0.45–1.72)	0.708	–	–
Anti‐dsDNA	0.60 (0.26–1.40)	0.237	–	–
Anti‐Sm	0.34 (0.13–0.89)	0.027	0.33 (0.13–0.86)	0.024
Anti‐Ro	0.84 (0.43–1.64)	0.603	–	–
Anti‐La	0.92 (0.32–2.70)	0.878	–	–
Anti‐RNP	0.60 (0.29–1.23)	0.163	–	–
Medications at induction				
Prednisolone dose	1.00 (0.98–1.03)	0.875	–	–
MMF	0.81 (0.37–1.77)	0.592	–	–
AZA	1.05 (0.35–3.16)	0.934	–	–
CNI	2.73 (0.51–14.6)	0.240	–	–
CTX	1.04 (0.14–7.62)	0.966	–	–
HCQ	1.23 (0.64–2.37)	0.541	–	–

* 24hUP, 24‐hour urine protein; AZA, azathioprine; C3, complement 3; C4, complement 4; CI, confidence interval; CNI, calcineurin inhibitor; CTX, cyclophosphamide; dsDNA, double‐stranded DNA; eGFR, estimated glomerular filtration rate; HCQ, hydroxychloroquine; ISN/RPS, International Society of Nephrology/Renal Pathology Society; LN, lupus nephritis; MMF, mycophenolate mofetil; OR, odds ratio; Ref, reference; SLE, systemic lupus erythematosus; Sm, Smith; UPCR, urine protein‐to‐creatinine ratio.

### Early LLDAS and CRR/PRR attainments can be applied to LN relapse risk stratification

Among 143 patients in the discovery cohort, 32 patients developed LN relapse. The median time to LN relapse was 2.8 years (IQR 1.5–5.0 years). Patients who developed LN relapse had more frequent nephrotic‐range proteinuria (LN relapse 31% vs no relapse 15%, *P* = 0.042) and hypoalbuminemia (LN relapse 29 g/L vs no relapse 32 g/L, *P* = 0.040) at baseline (Supplementary Table S2). The most frequent histologic subtypes at relapse were class IV ± V (20 of 32, 63%) and class III ± V (8 of 32, 25%). All patients were receiving glucocorticoids and maintenance immunosuppressive therapy at the time of LN relapse. Approximately 70% of patients were receiving hydroxychloroquine. Most patients had active serologic activity at the time of LN relapse (Supplementary Table S3).

**Figure 1:**
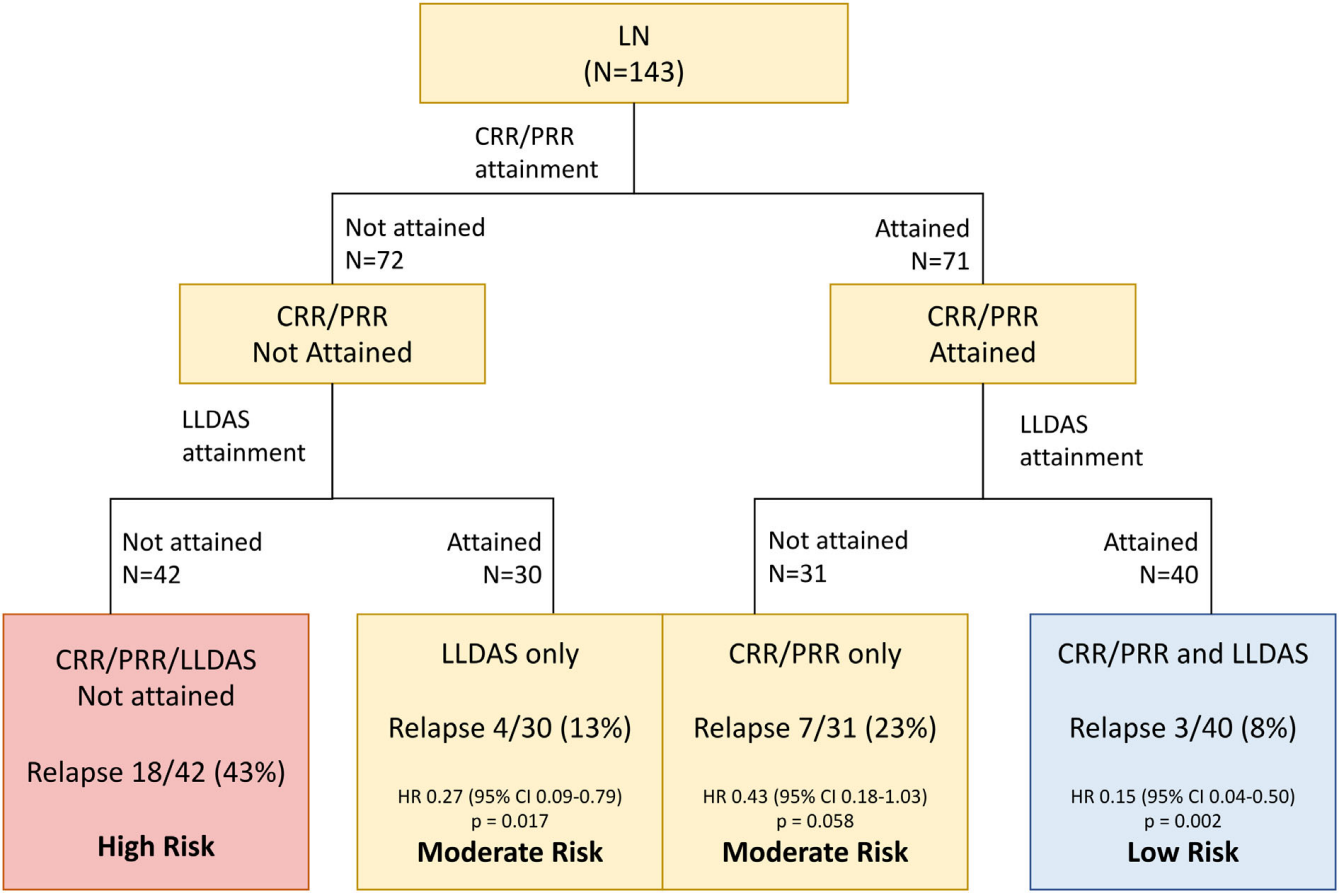
Relapse risk stratification based on treatment target attainment at 12 months. *Risk of renal relapse calculated using the “no response” group as the reference. CI, confidence interval; CRR, complete renal response; HR, hazard ratio; LLDAS, lupus low disease activity state; LN, lupus nephritis; PRR, partial renal response.

Patients were categorized into four groups stratified by treatment target attainment at 12 months to compare the risk of LN relapse (Figure 1). The first group (NR) comprised 42 patients who attained neither CRR/ PRR nor LLDAS; the second group (LLDAS‐only) comprised 30 patients who attained LLDAS without CRR/PRR at 12 months; the third group (CRR/PRR‐only) comprised 31 patients who attained CRR/PRR without LLDAS; the fourth group comprised 40 patients (CRR/PRR and LLDAS) who attained both targets. In the NR group, 18 of 42 (43%) patients developed LN relapse (“high risk”). Compared with the NR group, LLDAS‐only and CRR/PRR‐only groups had reduced risk of LN relapse (LLDAS‐only: hazard ratio [HR] 0.27, 95% CI 0.09–0.79, *P* = 0.017; CRR/PRR‐only: HR 0.43, 95% CI 0.18–1.03, *P* = 0.058; “moderate risk”). The lowest relapse risk was observed in the CRR/PRR and LLDAS group (HR 0.15, 95% CI 0.04–0.50; *P* = 0.002; “low risk”).

**Figure 2:**
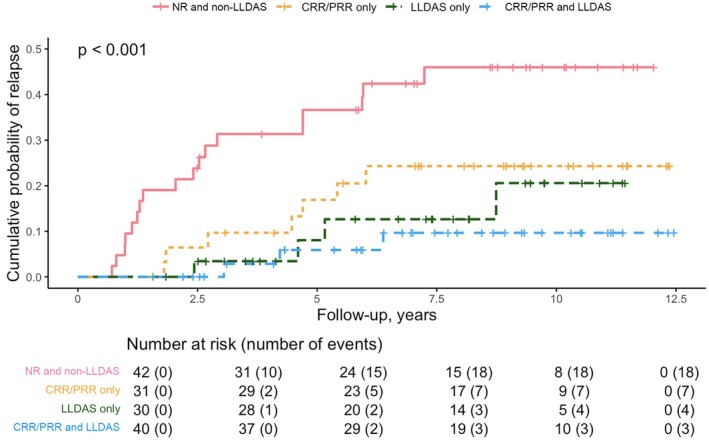
Cumulative risk of relapse over time stratified by renal response and LLDAS attainment. CRR, complete renal response; LLDAS, lupus low disease activity state; NR, no response; PRR, partial renal response. Color figure can be viewed in the online issue, which is available at http://onlinelibrary.wiley.com/doi/10.1002/acr.25611/abstract.

Kaplan‐Meier curves demonstrating the effects of CRR/PRR or LLDAS attainment on time to LN relapse were included in Figure 2. Patients were censored at the time of event, lost to follow‐up, and all‐cause mortality. Attainment of both CRR/PRR and LLDAS was associated with the best outcome compared with CRR/PRR alone, LLDAS alone, or failure to attain either target (Figure 2).

### LLDAS and CRR/PRR at 12 months reduce LN relapse risk by approximately 70%

Cox regression analysis was performed to identify independent factors of LN relapse in the discovery cohort (Table 3; Supplementary Table S3). In the univariate regression analysis, proteinuria ≥3 g at baseline (HR 2.22, 95% CI 1.02–4.58; *P* = 0.044), low serum albumin at baseline (HR 0.95, 95% CI 0.89–1.01; *P* = 0.094), anti‐Sm positivity (HR 2.15, 95% CI 0.99–4.68; *P* = 0.054), CRR/PRR attainment at 12 months (HR 0.40, 95% CI 0.19–0.85; *P* = 0.018), and LLDAS attainment at 12 months (HR 0.27, 95% CI 0.12–0.63; *P* = 0.002) were identified. Patients with DORIS remission had a lower frequency of LN relapse, but this did not reach statistical significance (HR 0.30, 95% CI 0.04–2.20; *P* = 0.236). Anti‐Sm positivity (HR 3.12, 95% CI 1.26–7.73; *P* = 0.014), CRR/PRR attainment at 12 months (HR 0.31, 95% CI 0.13–0.73; *P* = 0.007), and LLDAS attainment at 12 months (HR 0.38, 95% CI 0.16–0.91; *P* = 0.029) reduced LN relapse risk in multivariate analysis.

**Table 3:** Predictors of LN relapse in the discovery cohort*

Baseline characteristics	Univariable analysis		Multivariable analysis	
	HR (95% CI)	*P* value	HR (95% CI)	*P* value
Female sex	0.56 (0.20–1.68)	0.285	–	–
Age at SLE onset, years	1.00 (0.97–1.03)	0.910	–	–
Prior history of LN	1.27 (0.63–2.58)	0.503	–	–
ISN/RPS LN classes				
Class III (± V)	Ref	Ref	–	–
Class IV (± V)	0.99 (0.46–2.18)	0.993	–	–
Class V	0.55 (0.17–1.74)	0.306	–	–
24hUP (g) or UPCR (mg/mg)	1.12 (0.95–1.31)	0.186	–	–
≥3 g	2.22 (1.02–4.58)	0.044	1.78 (0.79–4.02)	0.165
Serum albumin, g/L	0.95 (0.89–1.01)	0.094	0.97 (0.91–1.04)	0.436
Serum creatinine, μmol/L	1.00 (0.99–1.01)	0.543	–	–
eGFR (mL/min/1.73m^2^)	1.00 (0.99–1.01)	0.709	–	–
Immunologic factors				
Low C3	0.95 (0.37–2.47)	0.916	–	–
Low C4	1.40 (0.67–2.91)	0.367	–	–
Anti‐dsDNA	1.04 (0.43–2.55)	0.710	–	–
Anti‐Sm	2.15 (0.99–4.68)	0.054	3.12 (1.26–7.73)	0.014
Anti‐Ro	1.12 (0.56–2.28)	0.742	–	–
Anti‐La	1.73 (0.66–4.50)	0.265	–	–
Anti‐RNP	1.15 (0.55–2.40)	0.711	–	–
Induction medication				
MMF	2.15 (0.76–6.14)	0.152	–	–
AZA	0.84 (0.26–2.75)	0.772	–	–
CNI	0.05 (0–58)	0.399	–	–
CTX	1.53 (0.21–11.27)	0.676	–	–
HCQ	0.76 (0.38–1.52)	0.430	–	–
Maintenance medications				
MMF	1.18 (0.51–2.73)	0.696	–	–
AZA	1.45 (0.60–3.53)	0.411	–	–
CNI	0.04 (0–13)	0.283	–	–
Treatment targets at 12 months				
CRR/PRR	0.40 (0.19–0.85)	0.018	0.31 (0.13–0.73)	0.007
LLDAS	0.27 (0.12–0.63)	0.002	0.38 (0.16–0.91)	0.029
DORIS remission	0.30 (0.04–2.20)	0.236	–	–

* 24hUP, 24‐hour urine protein; AZA, azathioprine; C3, complement 3; C4, complement 4; CI, confidence interval; CNI, calcineurin inhibitor; CRR/PRR, complete/partial renal response; CTX, cyclophosphamide; DORIS, Definition Of Remission In Systemic lupus erythematosus; dsDNA, double‐stranded DNA; eGFR, estimated glomerular filtration rate; HCQ, hydroxychloroquine; HR, hazard ratio; ISN/RPS, International Society of Nephrology/Renal Pathology Society; LLDAS, lupus low disease activity state; LN, lupus nephritis; MMF, mycophenolate mofetil; SLE, systemic lupus erythematosus; Sm, Smith; UPCR, urine protein‐to‐creatinine ratio.

The validation cohort was made up of 102 patients with LN from Pamela Youde Nethersole Eastern Hospital. Patients in the validation cohort had significantly higher baseline proteinuria, anti‐dsDNA titer, and serum creatinine levels. Follow‐up duration was longer in the validation cohort. Medication use varied between the discovery and validation cohorts. MMF induction and maintenance were less frequent in the validation cohort. The use of calcineurin inhibitors was more frequent in both induction and the maintenance treatment in the validation cohort. Azathioprine maintenance was more frequent in the validation cohort. Other details of the validation cohort are summarized in Table 1.

Cox regression analysis was performed to identify factors of LN relapse in the validation cohort. In the univariate analysis, serum albumin (HR 1.05, 95% CI 0.99–1.10; *P* = 0.090), CRR/PRR attainment (HR 0.41, 95% CI 0.18–0.92; *P* = 0.030) and LLDAS attainment (HR 0.41, 95% CI 0.19–0.87; *P* = 0.020) were identified. In the multivariate analysis, CRR/PRR attainment and LLDAS attainment reduced LN relapse (CRR/PPR attainment: HR 0.43, 95% CI 0.19–0.97; *P* = 0.043; LLDAS attainment: HR 0.40, 95% CI 0.19–0.86; *P* = 0.018) (Supplementary Tables S3 and S4).

### LLDAS attainment is associated with long‐term renal function preservation

**Figure 3:**
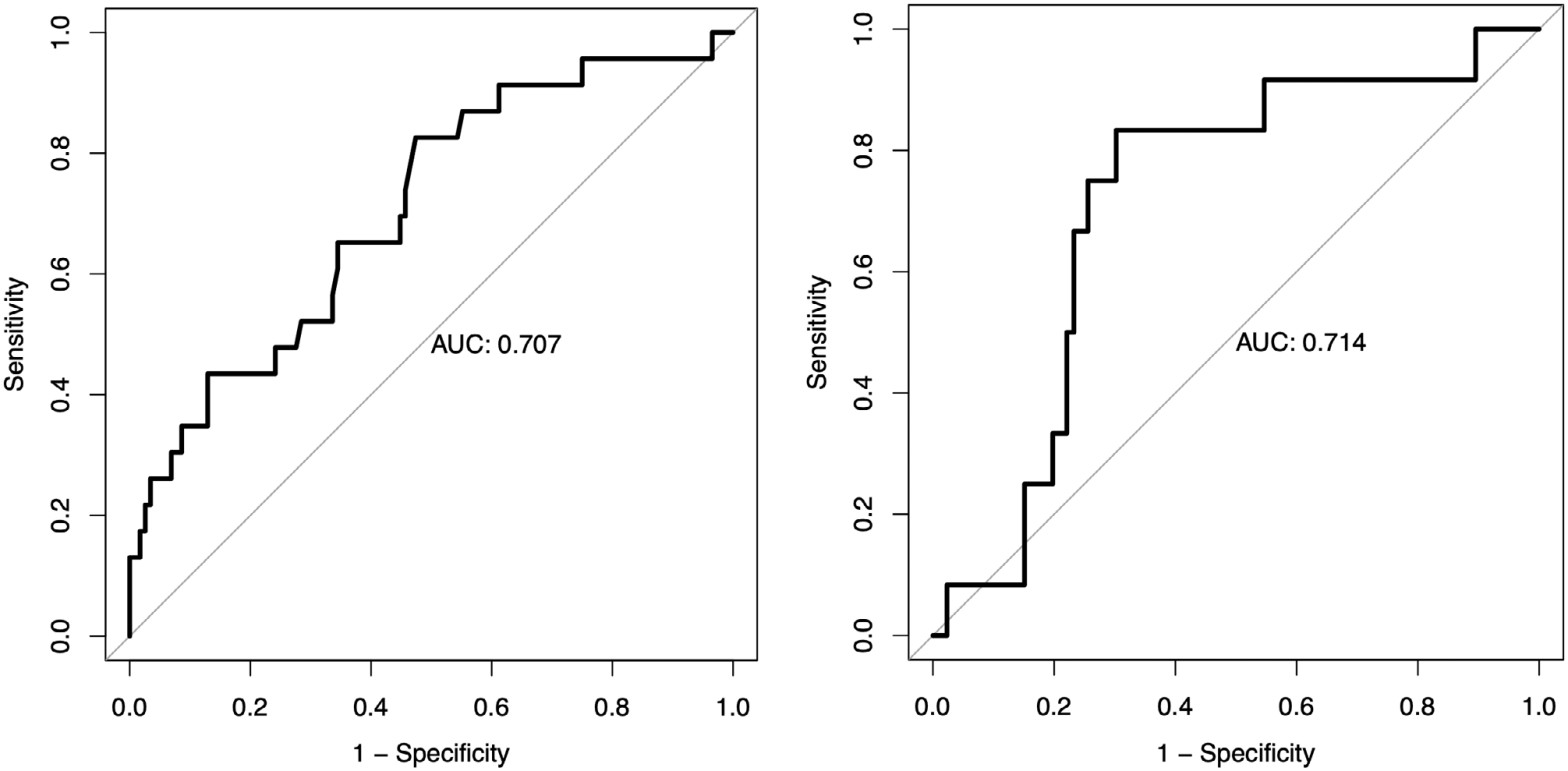
Receiver operating characteristic curve of LLDAS attainment for renal function deterioration in the (left) discovery cohort and (right) validation cohort. AUC, area under the curve.

Over a median follow‐up duration of 8.8 years, 25 of 143 (17%) patients developed renal function deterioration (defined as sustained deterioration with doubling of baseline serum creatinine) and 13 of 143 (9%) patients developed ESRD in the discovery cohort. The AUC of LLDAS attainment for predicting renal function deterioration was 0.71 in both the discovery and validation cohorts (Figure 3).

## DISCUSSION

To the best of our knowledge, this is the first study that evaluates the role of LLDAS and its usefulness in predicting the long‐term LN relapse and renal function preservation in patients with LN. LLDAS is a validated treatment target in SLE associated with reduced risks of disease relapse and organ damage. Our study demonstrates that LLDAS is an attainable and beneficial treatment target in patients with LN. The frequency of LLDAS attainment is comparable to that of CRR/PRR and is associated with a reduced risk of LN relapse. The attainment of LLDAS in addition to CRR/PPR confers a greater reduction in LN relapse risk compared with the attainment of either target alone. These findings suggest that LLDAS attainment is associated with renal function preservation.

Despite treatment advances in recent decades, ESRD remains an important complication and occurs in >10% of patients with LN.^19^, ^20^ The treatment goals of LN include disease remission, prevention of disease relapse, preservation of kidney function, and minimization of drug‐related toxicities.^21^, ^22^ The effectiveness of induction therapy is often assessed by surrogate parameters reflective of renal disease activity and damage, including serum creatinine, proteinuria, and urinary sediments. CRR/PRR remains the current recommended treatment target for LN. Despite the protective benefit of CRR/PRR attainment in long‐term renal outcomes, LN relapses may occur in up to half of those with LN in 10 years after the initial successful treatment.^3^, ^23^ Ongoing efforts are conducted in search of noninvasive prognostic biomarkers in LN.^24^ Urinary and serum markers have shown early promise, but their applications have been mostly limited to research settings only.

Multiple studies have evaluated the usefulness of LLDAS and its association with various outcome benefits, including prevention of relapse, reduced risk of organ damage accrual, better health‐related quality of life, and reduced risk of mortality. In addition to its role in defining a state of low disease activity in SLE, several studies have explored the potential application of LLDAS as a treatment target in SLE. A post hoc analysis of pooled data from the TULIP‐1 and TULIP‐2 trials demonstrated that LLDAS attainment was highly associated with British Isles Lupus Assessment Group‐based Composite Lupus Assessment and Systemic Lupus Erythematosus Responder Index (4) responses.^25^ Furthermore, anifrolumab treatment was associated with earlier, more frequent, and more prolonged and sustained LLDAS.^26^ Similarly, a post hoc analysis of data from the BLISS‐52 and BLISS‐76 trials showed that LLDAS was useful to discriminate responders to belimumab treatment.^27^ This study added evidence to the literature regarding LLDAS as a predictor of future relapse. However, the clinical application of LLDAS in LN remains to be explored. In a prospective study of 185 Chinese patients with SLE, nephritis‐related markers (proteinuria and serum creatinine) and C3 levels at recruitment negatively influenced the achievement of LLDAS.^11^ A similar observation was shown in a separate study of 107 White patients with SLE.^10^

SLE is a heterogeneous disease with interethnic differences in disease severity and treatment response, including more frequent and severe renal involvement among Asian patients.^28^, ^29^ One advantage of our study is the homogenous Chinese ethnicity among patients, which theoretically represents a more difficult to treat population. Furthermore, our study also showed a possible risk stratification approach based on treatment target attainment to potentially guide treatment decisions. Patients who fail to achieve CRR/PRR are known to be associated with a high risk of relapse and poor renal outcomes. Our results showed that LLDAS can be applied as an alternative treatment target with a comparable outcome on relapse prevention, and patients who attain both CRR/PRR and LLDAS represent a low‐risk group with the most significant relapse risk reduction. With the ROC curve analysis illustrating a satisfactory performance of LLDAS as a predictor, our findings demonstrate promise in the potential application of LLDAS as a treatment target and endpoint for future LN trials.

Our current study failed to show an association between DORIS remission and LN relapse risk reduction largely because of the small number of patients in DORIS remission at 12 months after active LN. Post hoc analysis of major clinical trials in patients with SLE also reflected the low DORIS remission rate in patients with recent disease flare.^30^ A longer time to remission is often observed compared with LLDAS, representing gradual improvement and a continuum of treatment target attainment in some patients.^31^ LLDAS may therefore have a unique role in the assessment of early treatment response. A larger cohort may be necessary to further evaluate the benefits of DORIS remission in patients with LN.

The presence of active extrarenal disease is common among patients with active LN.^32^ In our study, extrarenal disease activity occurred in almost half of the patients at baseline. By incorporating the assessment of extrarenal domains (such as the SLEDAI‐2K score), LLDAS has an advantage in capturing extrarenal disease activities compared with CRR/PRR. The potential benefit of LLDAS in patients with LN and extrarenal disease activity was shown in our analysis. Among patients with extrarenal disease activity at baseline, LLDAS predicted a greater reduction of relapse than CRR/PRR. This indicates that in patients with LN with extrarenal disease activity, one should not only aim for CRR/PRR in the renal domain, but also attempt to achieve LLDAS to ensure long‐term disease stability. Furthermore, the degree of immunosuppressive treatments in LN warrants a delicate balance, in which inadequate immunosuppression may predispose patients to relapse while oversuppression of the immune system can lead to excessive infective complications and toxicities.^22^ In this study, LLDAS and CRR/PRR showed a higher AUC than CRR/PRR alone, insinuating that LLDAS and CRR/PRR are feasible treatment targets in LN and the achievement of both targets are associated with the best outcomes on relapse prevention.

Interestingly, our study showed the association between the anti‐Sm autoantibody and the risk of relapse in patients with LN. Anti‐Sm is classically known to be a specific marker of SLE. Its role in patients with LN has yet to be elucidated. Previous studies showed its correlation with disease phenotypes and poorer renal outcomes.^33^, ^34^, ^35^ Another study identified anti‐Sm as a negative predictor for the attainment of LLDAS‐50 (defined as LLDAS for ≥50% of the time).^36^ Even though the current study included only a small number of patients with SLE and anti‐Sm positivity, a higher risk of LN relapse and a lower likelihood of LLDAS attainment were observed among this subgroup of patients. LLDAS was shown to be helpful in this subgroup of anti‐Sm–positive patients with LN in reducing the risk of relapse.

This study examined the relationship between treatment target attainment and LN relapse; future studies are needed to explore other clinical outcomes, including extrarenal flares and CKD progression. Only Chinse patients were included in our cohorts, and further studies are needed to confirm the generalizability for patients of various backgrounds. The 12‐month renal response included both CRR and PRR and their effect on subsequent renal outcomes were not evaluated individually. In our cohort, most patients who attained PRR at 12 months eventually attained proteinuric target below 500 to 700 mg per day, representing a subgroup of patients with slow proteinuric response. The gradual improvement may not be applicable to all patients. Therefore, it is essential to prioritize regular monitoring of clinical progress, particularly in patients exhibiting early partial response. Prospective studies are needed to ascertain the complementary benefit of LLDAS in LN.

This study provides evidence of LLDAS as an attainable treatment target in LN. The attainment of LLDAS alone or complementary to CRR/PRR was associated with a reduced risk of LN relapse.

## AUTHOR CONTRIBUTIONS

All authors contributed to at least one of the following manuscript preparation roles: conceptualization AND/OR methodology, software, investigation, formal analysis, data curation, visualization, and validation AND drafting or reviewing/editing the final draft. As corresponding author, Dr Chan confirms that all authors have provided the final approval of the version to be published and takes responsibility for the affirmations regarding article submission (eg, not under consideration by another journal), the integrity of the data presented, and the statements regarding compliance with institutional review board/Declaration of Helsinki requirements.

## Supporting information


**Disclosure form**.

**Supplementary Table S1:** Comparison of baseline characteristics between patients with or without LLDAS in the discovery cohort.
**Supplementary Table S2:** Comparison of baseline characteristics between patients with or without LN relapse in the discovery cohort.
**Supplementary Table S3:** Clinical and histological features at LN relapse of the discovery cohort
**Supplementary Table S4:** Predictors of LN renal relapse in the validation cohort

AC&R Journal Club
