# Supplementary material for: Reduction in Renal Relapse and Preservation of Long‐Term Kidney Function After Lupus Low Disease Activity in Patients With Lupus Nephritis
Source: Arthritis Care Res (Hoboken). 2025 Nov 21;78(2):227–36. doi: 10.1002/acr.25611 (PMC12919694; doi:10.1002/acr.25611)
Supplement: Supplementary file 2 — Supplementary Table S1: Comparison of baseline characteristics between patients with or without LLDAS in the discovery cohort. Supplementary Table S2: Comparison of baseline characteristics between patients with or without LN relapse in the discovery cohort. Supplementary Table S3: Clinical and histological features at LN relapse of the discovery cohort Supplementary Table S4: Predictors of LN renal relapse in the validation cohort [file ACR-78-227-s002.docx]

**Supplementary Table S1: Comparison of baseline characteristics between patients with or without LLDAS in the discovery cohort.**

| Baseline characteristics | LLDAS attained  (n=70) | LLDAS not attained  (n=73) |
| --- | --- | --- |
|  |  |  |
| Sex (female) | 65/70 (93%) | 66/73 (90%) |
| Age at SLE onset (years) | 26 (20-36) | 29 (20-35) |
| Prior history of LN | 33/70 (47%) | 41/73 (56%) |
| ISN/RPS LN classes |  |  |
| Class III (+/- V) | 19/70 (27%) | 19/73 (26%) |
| Class IV (+/- V) | 32/70 (46%) | 36/73 (49%) |
| Class V | 12/70 (17%) | 13/73 (18%) |
| 24huP(g) or UPCR (mg/mg) | 1.4 (1.2-2.1) | 1.7 (1.2-2.7) |
| ≥3g | 11/70 (16%) | 16/73 (22%) |
| Serum albumin (g/L) | 32 (29-36) | 31 (28-35) |
| Serum creatinine (μmol/L) | 63 (52-88) | 66 (54-86) |
| eGFR (mL/min/1.73m^2^) | 100 (67-123) | 97 (68-124) |
| Immunological factors |  |  |
| Low C3 | 56/70 (80%) | 66/73 (91%) |
| Low C4 | 41/70 (59%) | 45/73 (62%) |
| Anti-dsDNA | 54/70 (77%) | 62/73 (85%) |
| Anti-Sm | 7/66 (11%) | 18/70 (26%) |
| Anti-Ro | 31/66 (47%) | 36/70 (51%) |
| Anti-La | 7/66 (11%) | 8/70 (11%) |
| Anti-RNP | 18/66 (27%) | 27/70 (39%) |
| Medications at induction |  |  |
| Prednisolone dose | 40 (30-50) | 40 (30-48) |
| MMF | 53/70 (76%) | 58/73 (80%) |
| AZA | 7/70 (10%) | 7/73 (10%) |
| CNI | 5/70 (7%) | 2/73 (3%) |
| CTX | 2/70 (3%) | 2/73 (3%) |
| HCQ | 40/70 (57%) | 38/73 (52%) |

AZA= azathioprine; C3= complement 3; C4= complement 4; CNI= calcineurin inhibitors; CKD= chronic kidney disease; CTX= cyclophosphamide; eGFR= estimated glomerular filtration rate; GC=glucocorticoids; HCQ= hydroxychloroquine; ISN/RPS= International Society of Nephrology/Renal Pathology Society; LLDAS= lupus low disease activity state; LN= lupus nephritis; MMF= mycophenolate mofetil; RNP= ribonucleoprotein; SLE = systemic lupus erythematosus; Sm= smith; 24hUP= 24-hour urine protein, UPCR= urine protein to creatinine ratio.

**Supplementary Table S2: Comparison of baseline characteristics between patients with or without LN relapse in the discovery cohort.**

| Baseline characteristics | LN relapse  (n=32) | No LN relapse  (n=111) |
| --- | --- | --- |
|  |  |  |
| Sex (female) | 28/32 (88%) | 103/111 (93%) |
| Age at SLE onset (years) | 29 (18-37) | 27 (20-35) |
| Prior history of LN | 19/32 (59%) | 55/111 (50%) |
| ISN/RPS LN classes |  |  |
| Class III (+/- V) | 10/32 (33%) | 28/111 (25%) |
| Class IV (+/- V) | 17/32 (50%) | 51/111 (46%) |
| Class V | 4/32 (13%) | 21/111 (19%) |
| 24huP(g) or UPCR (mg/mg) | 2.0 (1.2-3.3) | 1.5 (1.2-2.2) |
| ≥3g | 10/32 (31%) | 17/111 (15%) |
| Serum albumin (g/L) | 29 (27-33) | 32 (29-36) |
| Serum creatinine (μmol/L) | 65 (56-87) | 64 (52-88) |
| eGFR (mL/min/1.73m^2^) | 99 (66-114) | 98 (67-124) |
| Immunological factors |  |  |
| Low C3 | 27/32 (84%) | 95/111 (86%) |
| Low C4 | 21/32 (66%) | 65/111 (59%) |
| Anti-dsDNA | 26/32 (81%) | 90/111 (81%) |
| Anti-Sm | 9/31 (31%) | 16/105 (15%) |
| Anti-Ro | 16/31 (52%) | 52/105 (49%) |
| Anti-La | 5/31 (16%) | 10/105 (10%) |
| Anti-RNP | 11/31 (35%) | 34/105 (32%) |
| Induction medication |  |  |
| MMF | 28/32 (88%) | 83/111 (75%) |
| AZA | 3/32 (9%) | 11/111 (10%) |
| CNI | 0/32 (0%) | 7/111 (6%) |
| CTX | 1/32 (3%) | 3/111 (3%) |
| HCQ | 15/32 (47%) | 63/111 (57%) |
| Maintenance medications |  |  |
| MMF | 25/32 (78%) | 83/111 (75%) |
| AZA | 6/32 (19%) | 13/111 (12%) |
| CNI | 0/32 (0%) | 11/111 (10%) |
| Treatment targets at 12-month |  |  |
| CRR/PRR | 10/32 (31%) | 61/111 (55%) |
| LLDAS | 7/32 (22%) | 63/111 (57%) |
| DORIS remission | 1/32 (3%) | 14/111 (13%) |

AZA= azathioprine; C3= complement 3; C4= complement 4; CNI= calcineurin inhibitors; CKD= chronic kidney disease; CRR= complete renal response; CTX= cyclophosphamide; eGFR= estimated glomerular filtration rate; GC=glucocorticoids; HCQ= hydroxychloroquine; ISN/RPS= International Society of Nephrology/Renal Pathology Society; LLDAS= lupus low disease activity state; LN= lupus nephritis; MMF= mycophenolate mofetil; PRR= partial renal response; RNP= ribonucleoprotein; SLE = systemic lupus erythematosus; Sm= smith; 24hUP= 24-hour urine protein, UPCR= urine protein to creatinine ratio.

**Supplementary Table S3: Clinical and histological features at LN relapse of the discovery cohort**

| **Clinical characteristics** |  |
| --- | --- |
| Sex (female) | 27/30 (90%) |
| Time to relapse (years) | 3.0 (1.8-5.2) |
| ISN/RPS LN classification |  |
| Class III (+/- V) | 7/30 (23%) |
| Class IV (+/- V) | 19/30 (63%) |
| Class V | 2/30 (7%) |
| 24huP(g) or UPCR (mg/mg) | 1.8 (1.2-4.2) |
| Serum albumin (g/L) | 34 (27-38) |
| Serum creatinine (μmol/L) | 73 (54-105) |
| eGFR (mL/min/1.73m^2^) | 81 (53-110) |
| CKD categories |  |
| CKD1 | 13/30 (43%) |
| CKD2 | 8/30 (27%) |
| CKD3 | 7/30 (23%) |
| CKD4 | 2/30 (7%) |
| CKD5 | 0/30 (0%) |
| Presence of active serology |  |
| Low C3 | 23/30 (77%) |
| Low C4 | 12/30 (40%) |
| Low C3 or C4 | 23/30 (77%) |
| Elevated anti-dsDNA | 21/30 (70%) |
| Medication at LN relapse |  |
| GC | 30/30 (100%) |
| MMF | 23/30 (77%) |
| AZA | 3/30 (10%) |
| CNI | 4/30 (13%) |
| HCQ | 21/30 (70%) |

AZA= azathioprine; C3= complement 3; C4= complement 4; CNI= calcineurin inhibitors; CKD= chronic kidney disease; CTX= cyclophosphamide; eGFR= estimated glomerular filtration rate; GC=glucocorticoids; HCQ= hydroxychloroquine; ISN/RPS= International Society of Nephrology/Renal Pathology Society; LN= lupus nephritis; MMF= mycophenolate mofetil; 24hUP= 24-hour urine protein, UPCR= urine protein to creatinine ratio.

**Supplementary Table S4: Predictors of LN renal relapse in the validation cohort**

| Baseline characteristics | LN relapse  (n=28) | No LN relapse  (n=74) | Univariable analysis | | Multivariable analysis | |
| --- | --- | --- | --- | --- | --- | --- |
|  |  |  | HR (95%CI) | p -value | HR (95%CI) | p -value |
| Sex (female) | 27 (96%) | 62 (84%) | 5.13 (0.68-38.46) | 0.113 |  |  |
| Age at SLE onset (years) | 31 (16-39) | 30 (19-39) | 1.00 (0.97-1.03) | 0.858 |  |  |
| ISN/RPS LN classes |  |  |  |  |  |  |
| Class III (+/- V) | 8 (29%) | 25 (16%) | ref | ref |  |  |
| Class IV (+/- V) | 11 (39%) | 29 (39%) | 1.25 (0.48-3.24) | 0.645 |  |  |
| Class V | 5 (18%) | 8 (11%) | 1.76 (0.55-5.61) | 0.340 |  |  |
| 24huP(g) or UPCR (mg/mg) |  |  | 0.87 (0.70-1.09) | 0.215 |  |  |
| ≥3g | 8 (29%) | 26 (35%) | 0.76 (0.33-1.73) | 0.507 |  |  |
| Serum albumin (g/L) | 31 (25-38) | 31 (26-34) | 1.05 (0-99-1.10) | 0.090 | 1.01 (0.99-1.12) | 0.091 |
| Serum creatinine (μmol/L) | 67 (55-96) | 70 (61-93) | 1.00 (0.99-1.01) | 0.528 |  |  |
| eGFR (mL/min/1.73m^2^) | 93 (60-127) | 91 (70-108) | 1.00 (0.99-1.01) | 0.810 |  |  |
| Immunological factors |  |  |  |  |  |  |
| Low C3/C4 | 25 (89%) | 65 (96%) | 0.83 (0.25-2.78) | 0.767 |  |  |
| Anti-dsDNA | 27 (96%) | 66 (89%) | 2.60 (0.35-19.16) | 0.350 |  |  |
| Anti-Sm | 4 (14%) | 8 (11%) | 1.19 (0.40-3.57) | 0.752 |  |  |
| Anti-Ro | 10 (36%) | 36 (49%) | 0.68 (0.31-1.49) | 0.334 |  |  |
| Anti-La | 3 (11%) | 5 (7%) | 1.62 (0.49-5.39) | 0.434 |  |  |
| Anti-RNP | 7 (25%) | 16 (22%) | 1.50 (0.63-3.60) | 0.359 |  |  |
| Induction medication |  |  |  |  |  |  |
| MMF | 17 (61%) | 49 (66%) | 0.86 (0.40-1.84) | 0.691 |  |  |
| AZA | 4 (14%) | 13 (18%) | 0.74 (0.25-2.18) | 0.587 |  |  |
| CNI | 4 (14%) | 9 (12%) | 1.45 (0.50-4.22) | 0.493 |  |  |
| CTX | 1 (4%) | 4 (5%) | 0.93 (0.13-6.86) | 0.943 |  |  |
| HCQ | 21 (75%) | 50 (58%) | 1.38 (0.58-3.30) | 0.465 |  |  |
| Maintenance medications |  |  |  |  |  |  |
| MMF | 11 (39%) | 38 (51%) | 0.82 (0.38-1.76) | 0.608 |  |  |
| AZA | 8 (29%) | 23 (31%) | 0.76 (0.33-1.72) | 0.503 |  |  |
| CNI | 7 (25%) | 13 (18%) | 1.53 (0.64-3.63) | 0.338 |  |  |
| Treatment targets at 12-month |  |  |  |  |  |  |
| CRR/PRR | 11 (39%) | 40 (54%) | 0.41 (0.18-0.92) | 0.030 | 0.43 (0.19-0.97) | 0.043 |
| LLDAS | 12 (43%) | 51 (69%) | 0.41 (0.19-0.87) | 0.020 | 0.40 (0.19-0.86) | 0.018 |

AZA= azathioprine; C3= complement 3; C4= complement 4; CNI= calcineurin inhibitors; CKD= chronic kidney disease; CRR= complete renal response; CTX= cyclophosphamide; eGFR= estimated glomerular filtration rate; GC=glucocorticoids; HCQ= hydroxychloroquine; ISN/RPS= International Society of Nephrology/Renal Pathology Society; LLDAS= lupus low disease activity state; LN= lupus nephritis; MMF= mycophenolate mofetil; PRR= partial renal response; RNP= ribonucleoprotein; SLE = systemic lupus erythematosus; Sm= smith; 24hUP= 24-hour urine protein, UPCR= urine protein to creatinine ratio.
