# Supplementary material for: Reduction in Renal Relapse and Preservation of Long‐Term Kidney Function After Lupus Low Disease Activity in Patients With Lupus Nephritis
Source: Arthritis Care Res (Hoboken). 2025 Nov 21;78(2):227–36. doi: 10.1002/acr.25611 (PMC12919694; doi:10.1002/acr.25611)
Supplement: Supplementary file 3 — AC&R Journal Club [file ACR-78-227-s003.pptx]

## Slide 1
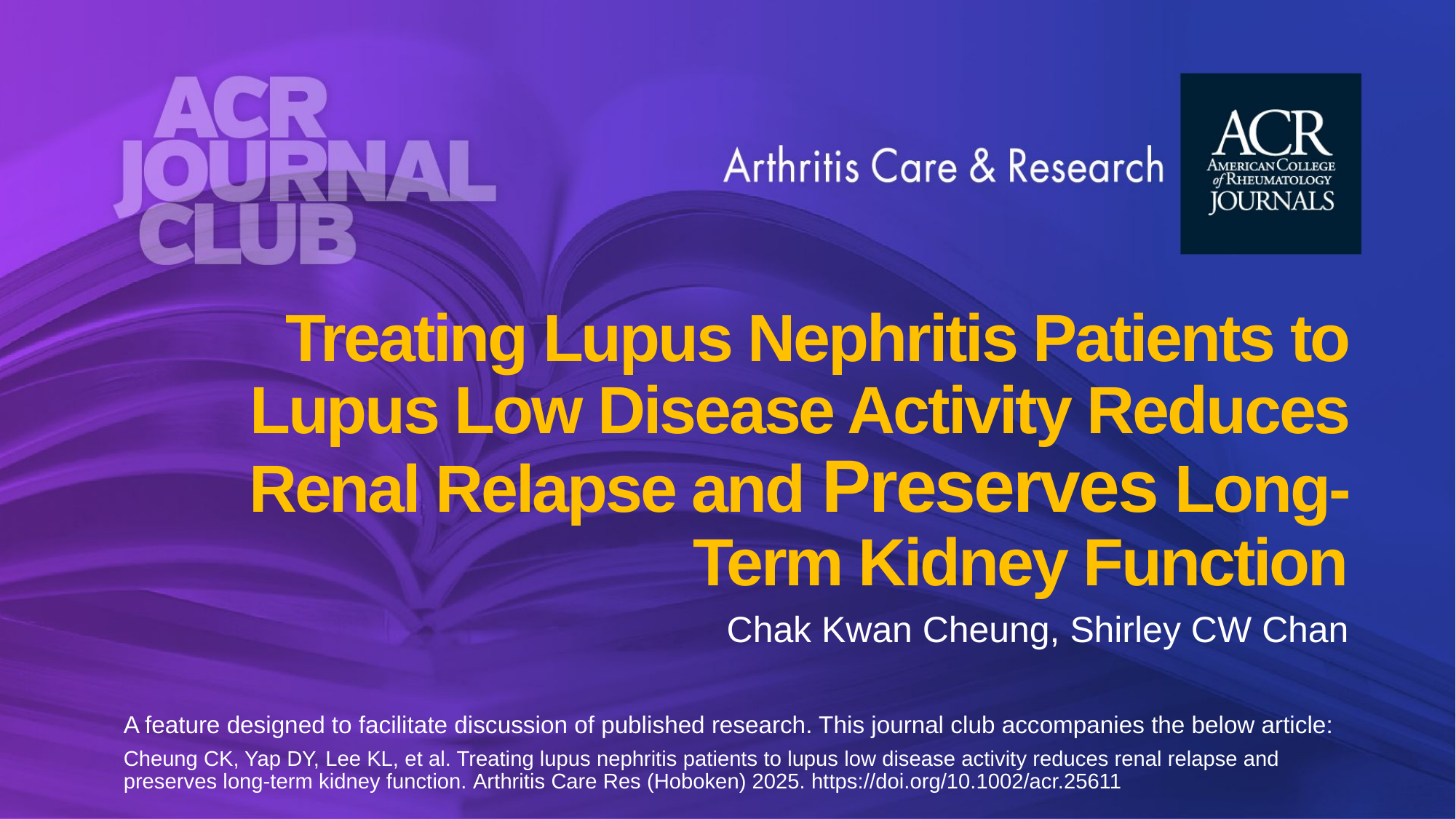

# Treating Lupus Nephritis Patients to Lupus Low Disease Activity Reduces Renal Relapse and Preserves Long-Term Kidney Function
Chak Kwan Cheung, Shirley CW Chan
A feature designed to facilitate discussion of published research. This journal club accompanies the below article:
Cheung CK, Yap DY, Lee KL, et al. Treating lupus nephritis patients to lupus low disease activity reduces renal relapse and preserves long-term kidney function. Arthritis Care Res (Hoboken) 2025. https://doi.org/10.1002/acr.25611

## Slide 2
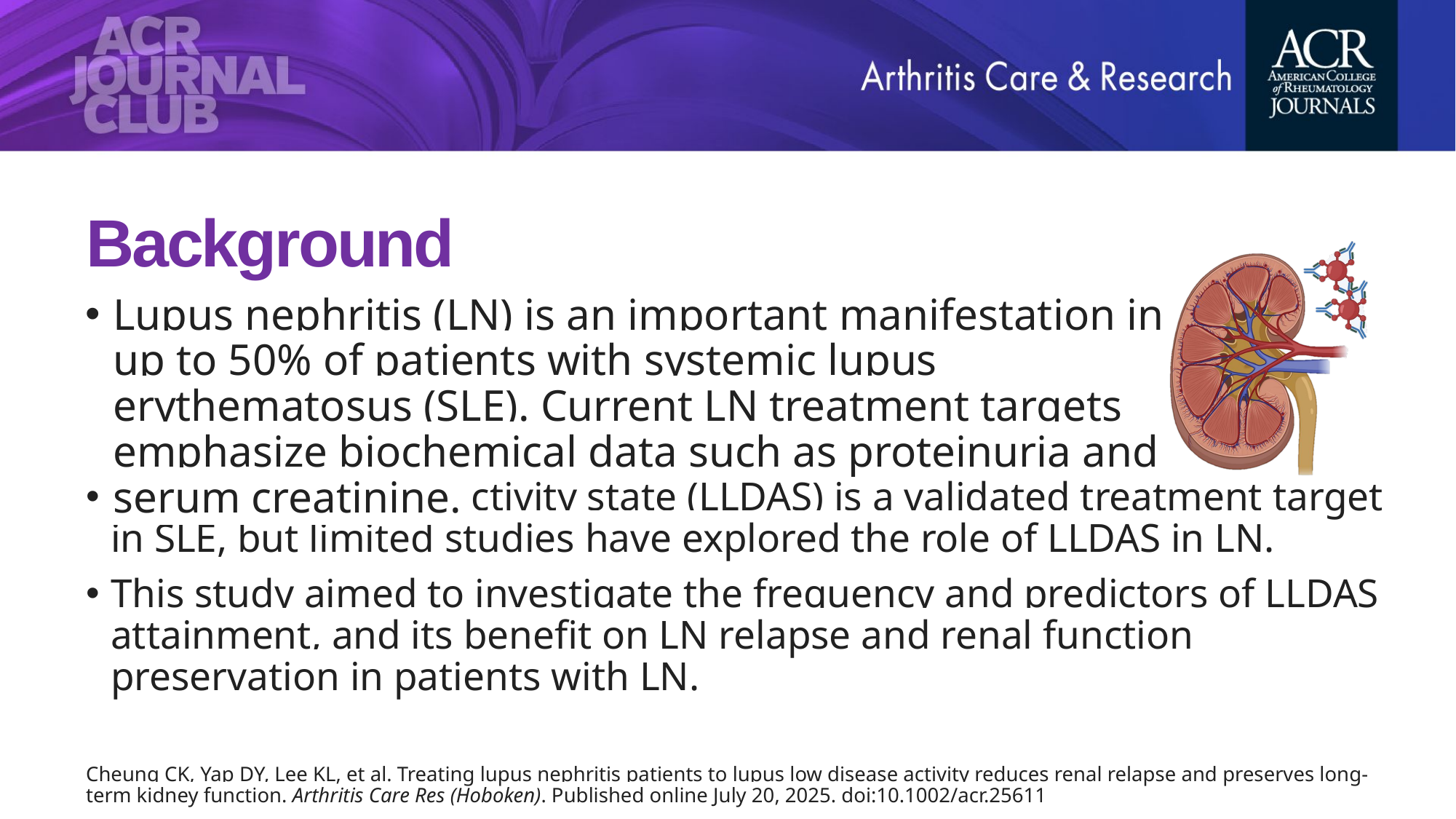

# Background
Lupus nephritis (LN) is an important manifestation in up to 50% of patients with systemic lupus erythematosus (SLE). Current LN treatment targets emphasize biochemical data such as proteinuria and serum creatinine.
Lupus low disease activity state (LLDAS) is a validated treatment target in SLE, but limited studies have explored the role of LLDAS in LN.
This study aimed to investigate the frequency and predictors of LLDAS attainment, and its benefit on LN relapse and renal function preservation in patients with LN.
Cheung CK, Yap DY, Lee KL, et al. Treating lupus nephritis patients to lupus low disease activity reduces renal relapse and preserves long-term kidney function. Arthritis Care Res (Hoboken). Published online July 20, 2025. doi:10.1002/acr.25611

## Slide 3
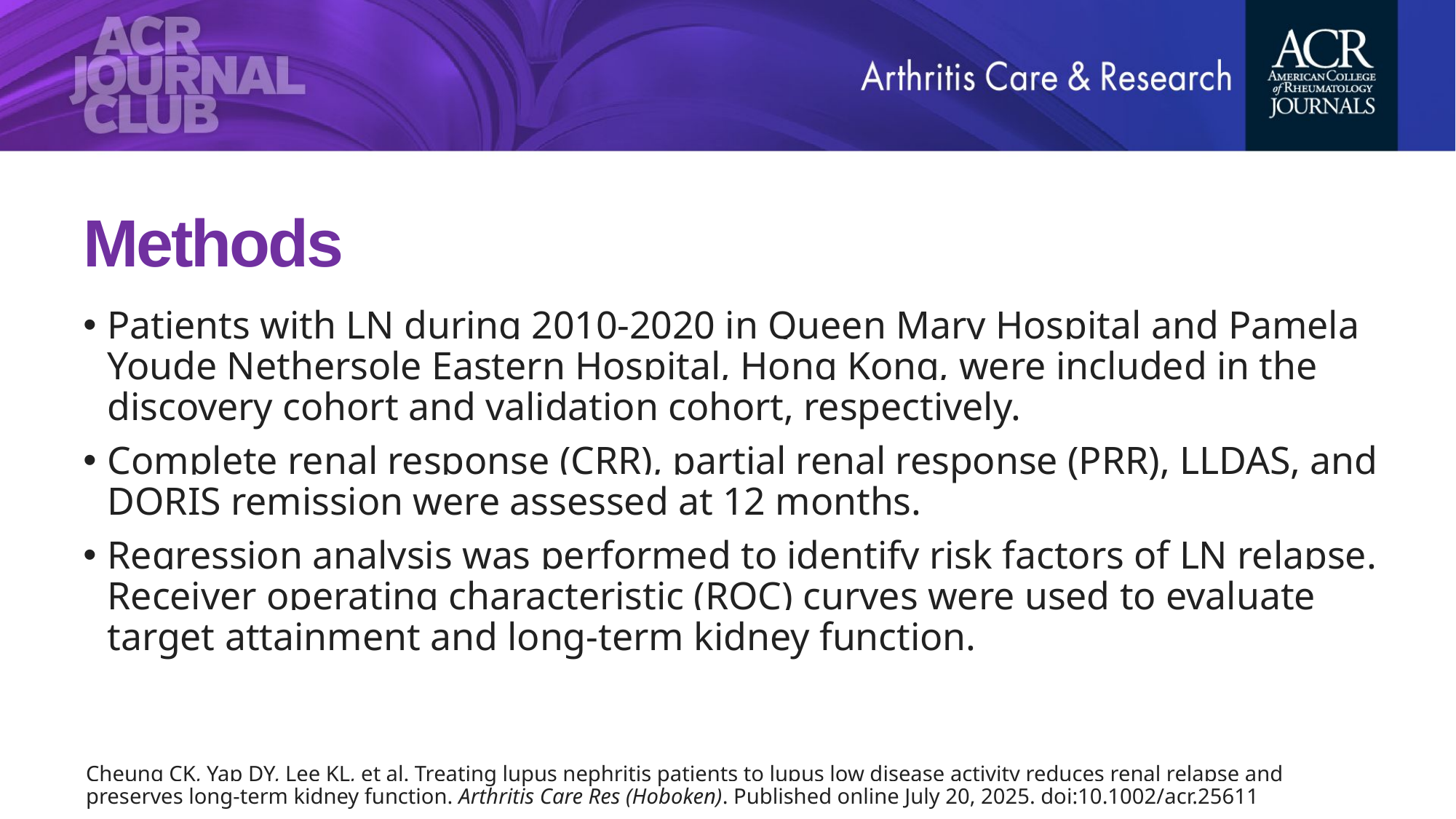

# Methods
Patients with LN during 2010-2020 in Queen Mary Hospital and Pamela Youde Nethersole Eastern Hospital, Hong Kong, were included in the discovery cohort and validation cohort, respectively.
Complete renal response (CRR), partial renal response (PRR), LLDAS, and DORIS remission were assessed at 12 months.
Regression analysis was performed to identify risk factors of LN relapse. Receiver operating characteristic (ROC) curves were used to evaluate target attainment and long-term kidney function.
Cheung CK, Yap DY, Lee KL, et al. Treating lupus nephritis patients to lupus low disease activity reduces renal relapse and preserves long-term kidney function. Arthritis Care Res (Hoboken). Published online July 20, 2025. doi:10.1002/acr.25611

## Slide 4
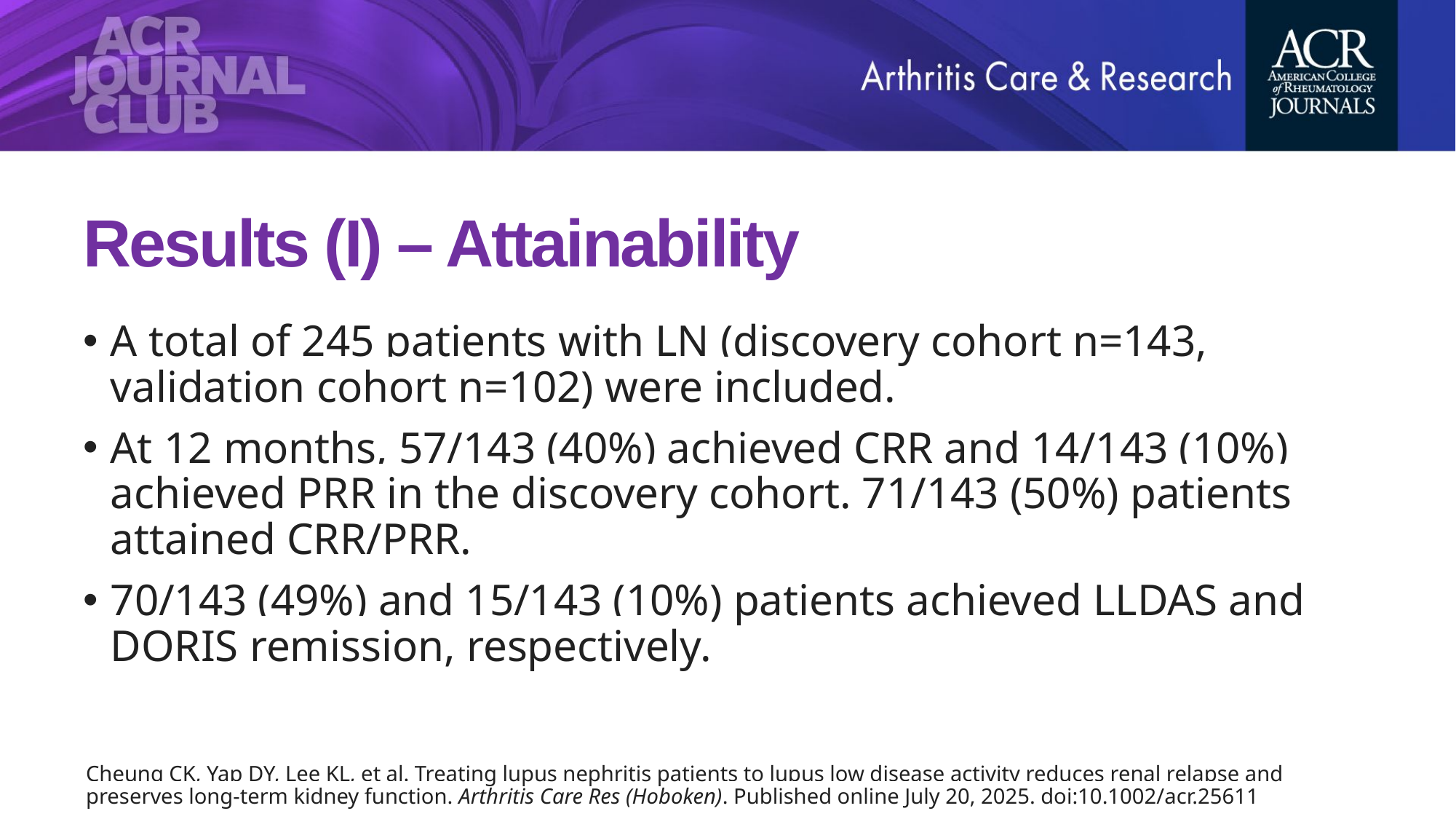

# Results (I) – Attainability
A total of 245 patients with LN (discovery cohort n=143, validation cohort n=102) were included.
At 12 months, 57/143 (40%) achieved CRR and 14/143 (10%) achieved PRR in the discovery cohort. 71/143 (50%) patients attained CRR/PRR.
70/143 (49%) and 15/143 (10%) patients achieved LLDAS and DORIS remission, respectively.
Cheung CK, Yap DY, Lee KL, et al. Treating lupus nephritis patients to lupus low disease activity reduces renal relapse and preserves long-term kidney function. Arthritis Care Res (Hoboken). Published online July 20, 2025. doi:10.1002/acr.25611

## Slide 5
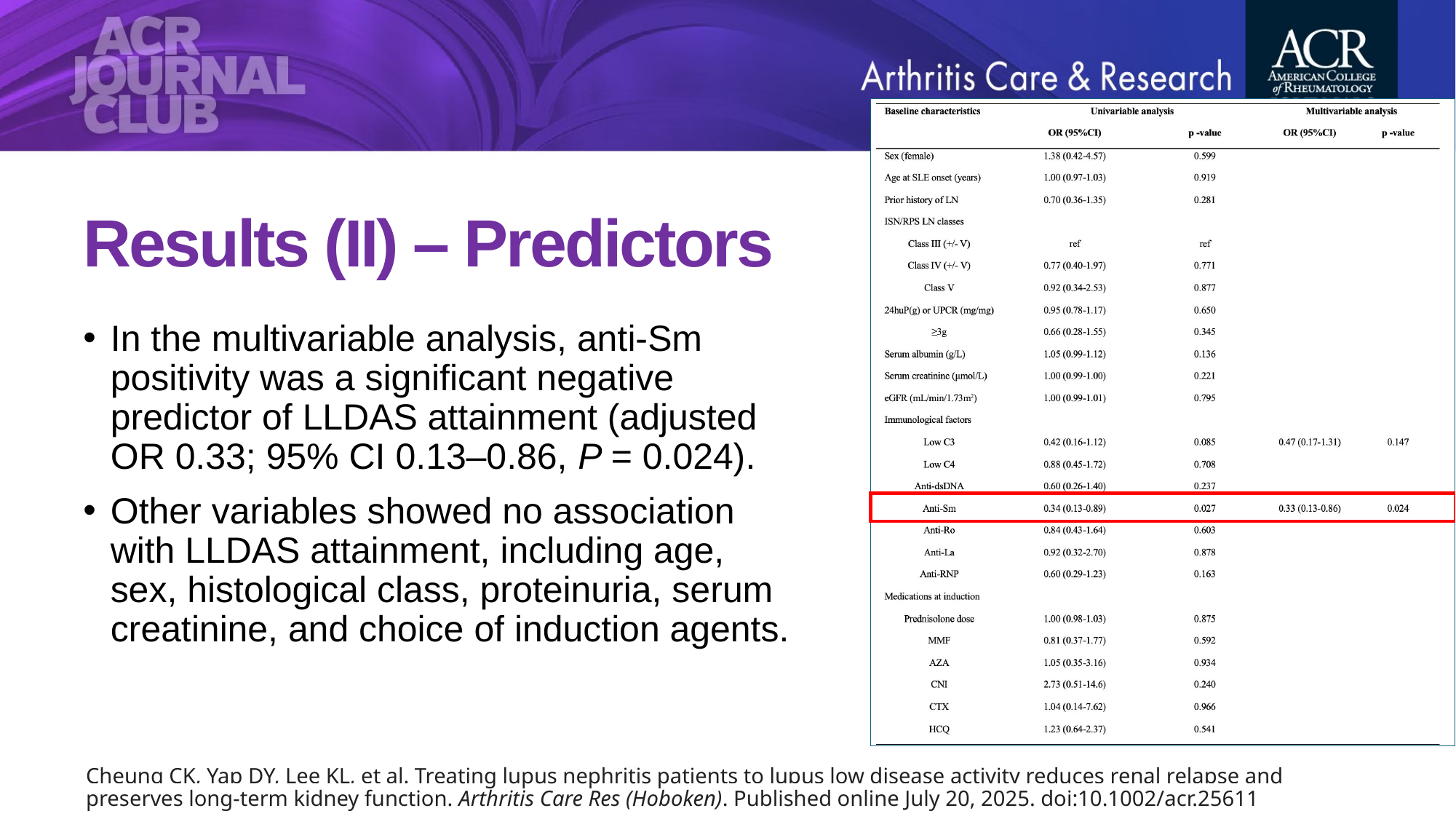

# Results (II) – Predictors
In the multivariable analysis, anti-Sm positivity was a significant negative predictor of LLDAS attainment (adjusted OR 0.33; 95% CI 0.13–0.86, P = 0.024).
Other variables showed no association with LLDAS attainment, including age, sex, histological class, proteinuria, serum creatinine, and choice of induction agents.
Cheung CK, Yap DY, Lee KL, et al. Treating lupus nephritis patients to lupus low disease activity reduces renal relapse and preserves long-term kidney function. Arthritis Care Res (Hoboken). Published online July 20, 2025. doi:10.1002/acr.25611

## Slide 6
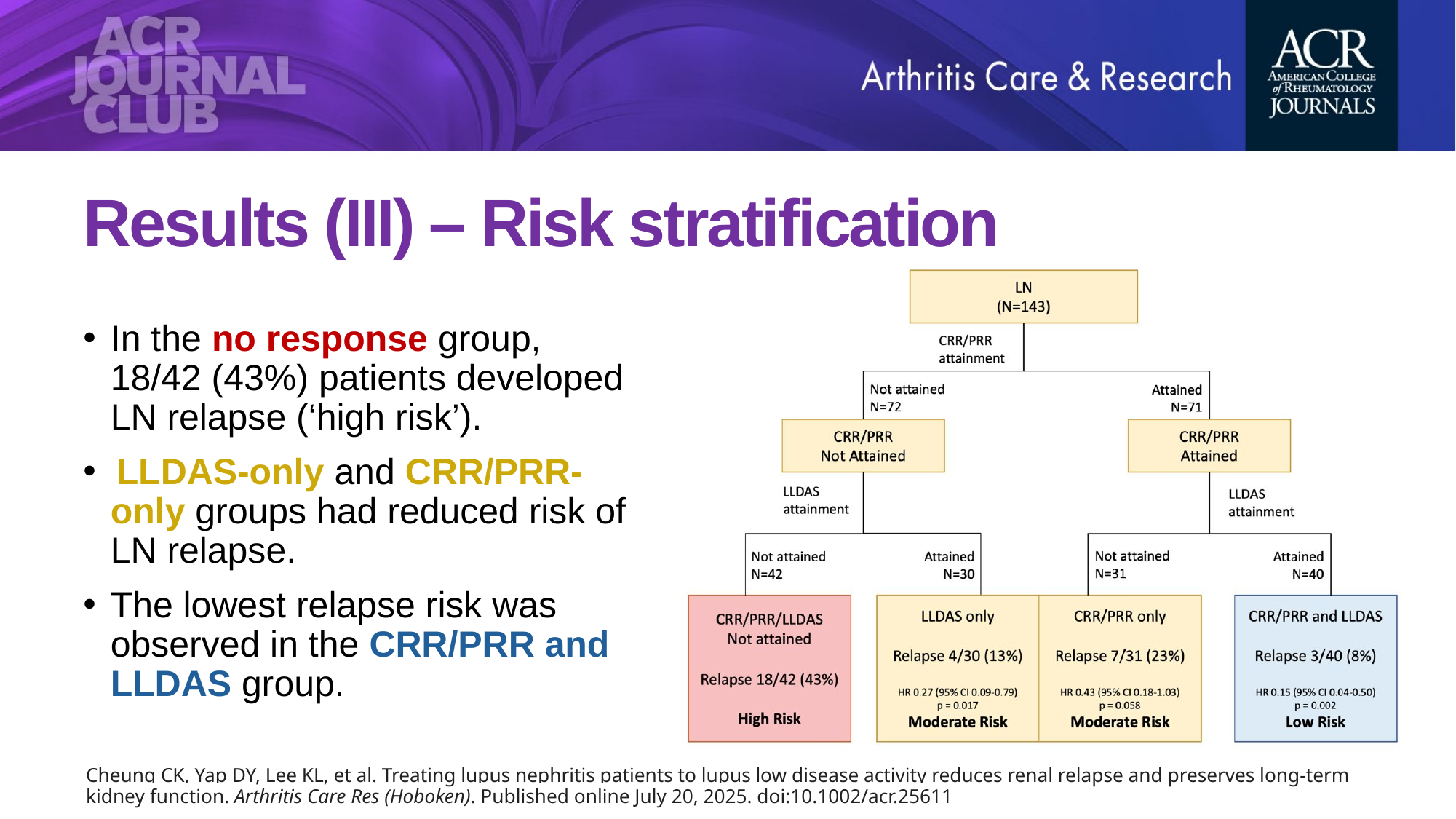

# Results (III) – Risk stratification
In the no response group, 18/42 (43%) patients developed LN relapse (‘high risk’).
 LLDAS-only and CRR/PRR-only groups had reduced risk of LN relapse.
The lowest relapse risk was observed in the CRR/PRR and LLDAS group.
Cheung CK, Yap DY, Lee KL, et al. Treating lupus nephritis patients to lupus low disease activity reduces renal relapse and preserves long-term kidney function. Arthritis Care Res (Hoboken). Published online July 20, 2025. doi:10.1002/acr.25611

## Slide 7
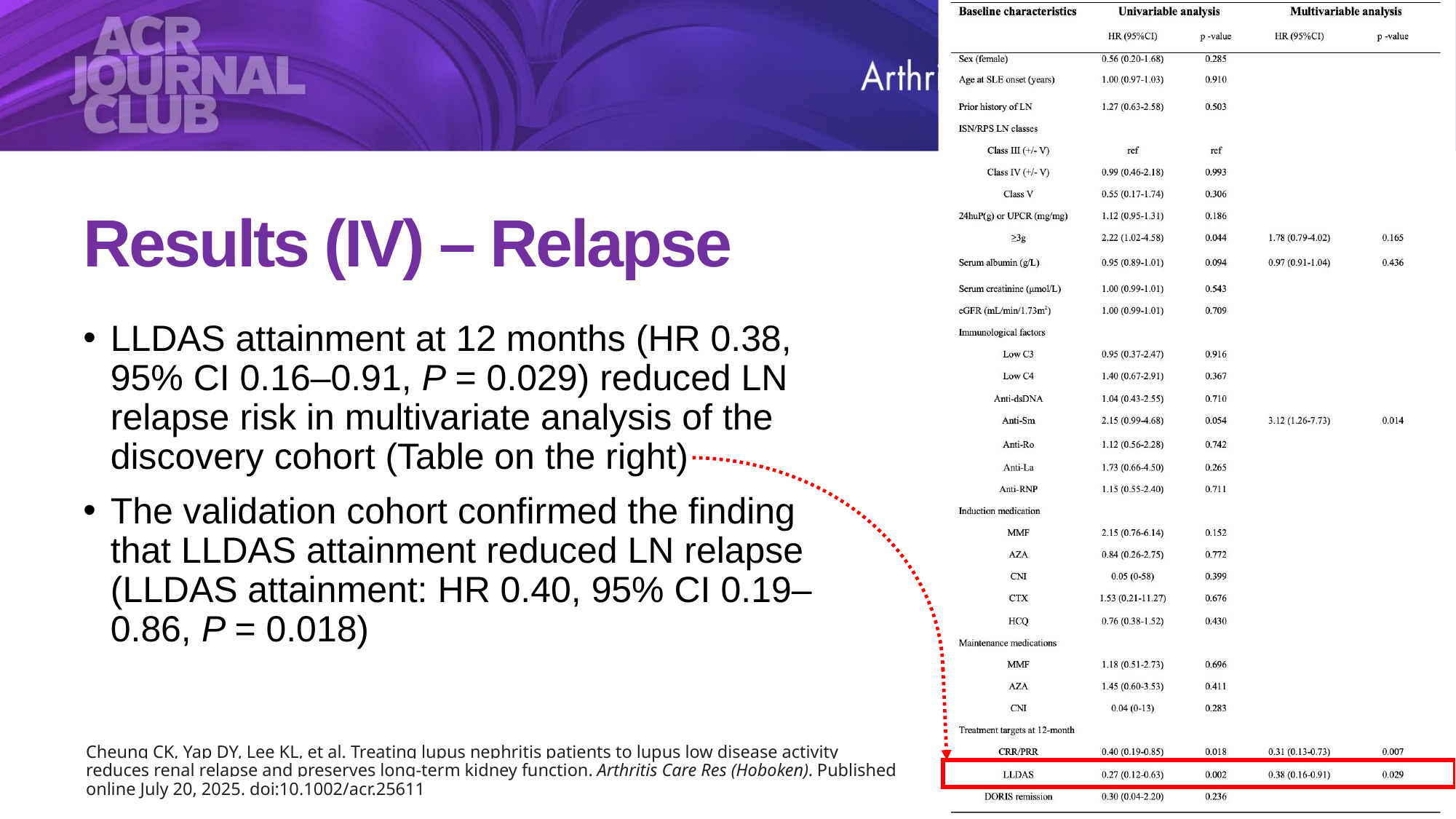

# Results (IV) – Relapse
LLDAS attainment at 12 months (HR 0.38, 95% CI 0.16–0.91, P = 0.029) reduced LN relapse risk in multivariate analysis of the discovery cohort (Table on the right)
The validation cohort confirmed the finding that LLDAS attainment reduced LN relapse (LLDAS attainment: HR 0.40, 95% CI 0.19–0.86, P = 0.018)
Cheung CK, Yap DY, Lee KL, et al. Treating lupus nephritis patients to lupus low disease activity reduces renal relapse and preserves long-term kidney function. Arthritis Care Res (Hoboken). Published online July 20, 2025. doi:10.1002/acr.25611

## Slide 8
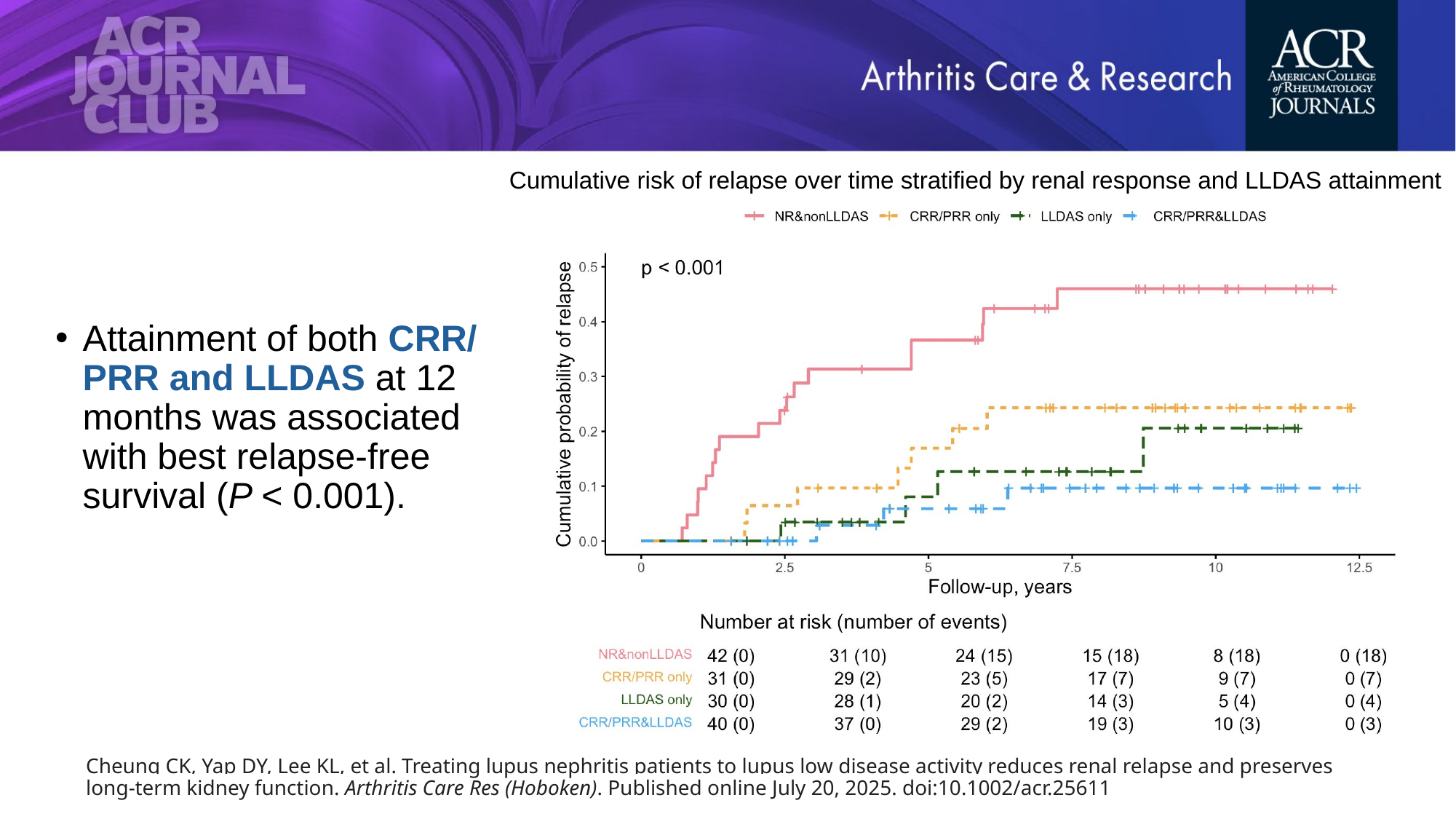

Cumulative risk of relapse over time stratified by renal response and LLDAS attainment
Attainment of both CRR/ PRR and LLDAS at 12 months was associated with best relapse-free survival (P < 0.001).
Cheung CK, Yap DY, Lee KL, et al. Treating lupus nephritis patients to lupus low disease activity reduces renal relapse and preserves long-term kidney function. Arthritis Care Res (Hoboken). Published online July 20, 2025. doi:10.1002/acr.25611

## Slide 9
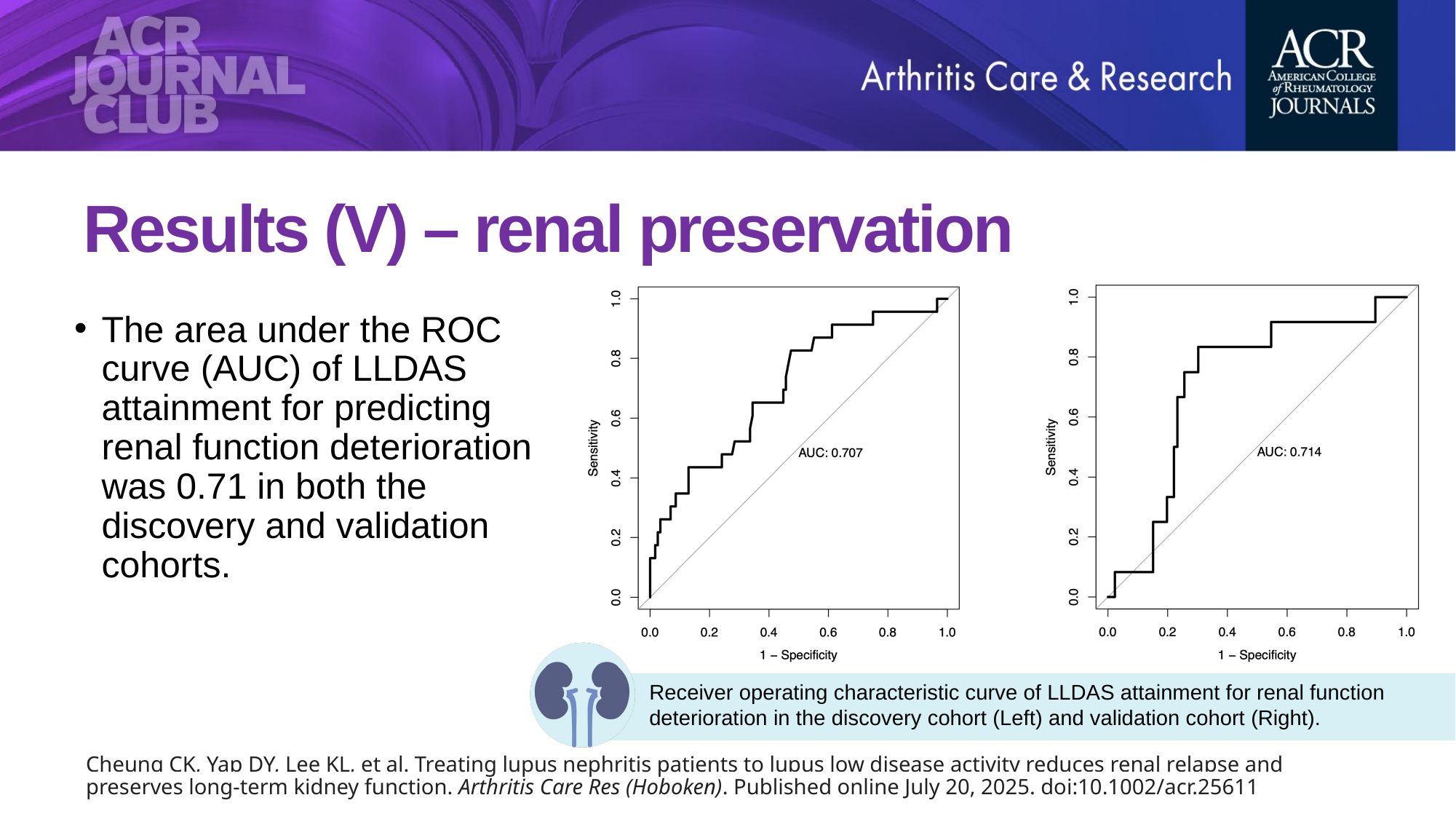

# Results (V) – renal preservation
The area under the ROC curve (AUC) of LLDAS attainment for predicting renal function deterioration was 0.71 in both the discovery and validation cohorts.
Receiver operating characteristic curve of LLDAS attainment for renal function deterioration in the discovery cohort (Left) and validation cohort (Right).
Cheung CK, Yap DY, Lee KL, et al. Treating lupus nephritis patients to lupus low disease activity reduces renal relapse and preserves long-term kidney function. Arthritis Care Res (Hoboken). Published online July 20, 2025. doi:10.1002/acr.25611

## Slide 10
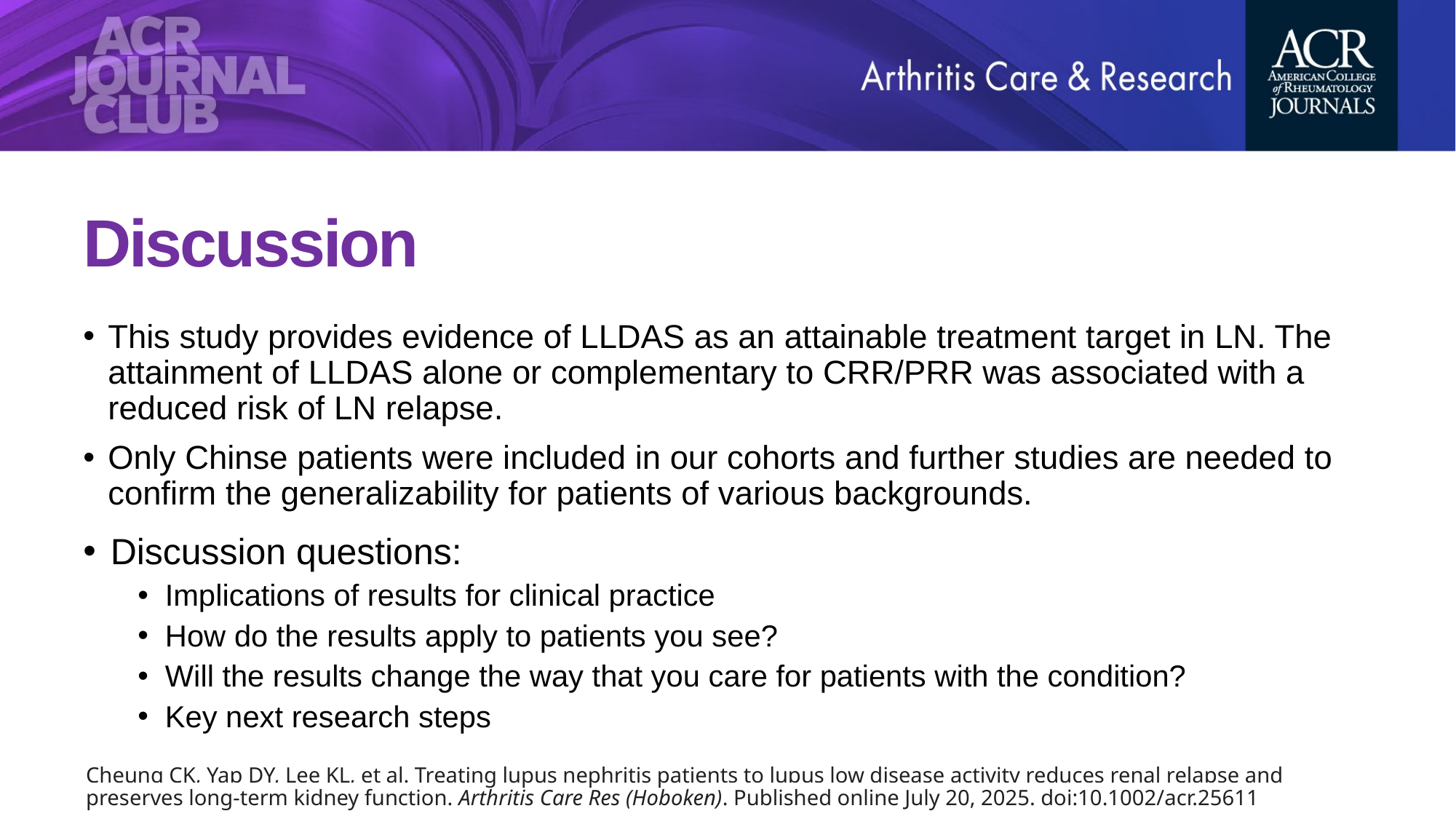

# Discussion
This study provides evidence of LLDAS as an attainable treatment target in LN. The attainment of LLDAS alone or complementary to CRR/PRR was associated with a reduced risk of LN relapse.
Only Chinse patients were included in our cohorts and further studies are needed to confirm the generalizability for patients of various backgrounds.
Discussion questions:
Implications of results for clinical practice
How do the results apply to patients you see?
Will the results change the way that you care for patients with the condition?
Key next research steps
Cheung CK, Yap DY, Lee KL, et al. Treating lupus nephritis patients to lupus low disease activity reduces renal relapse and preserves long-term kidney function. Arthritis Care Res (Hoboken). Published online July 20, 2025. doi:10.1002/acr.25611
